# Pseudo‐spiral sampling and compressed sensing reconstruction provides flexibility of temporal resolution in accelerated aortic 4D flow MRI: A comparison with k‐t principal component analysis

**DOI:** 10.1002/nbm.4255

**Published:** 2020-01-20

**Authors:** Lukas M. Gottwald, Eva S. Peper, Qinwei Zhang, Bram F. Coolen, Gustav J. Strijkers, Aart J. Nederveen, Pim van Ooij

**Affiliations:** ^1^ Department of Radiology and Nuclear Medicine, Amsterdam University Medical Centers University of Amsterdam the Netherlands; ^2^ Department of Biomedical Engineering and Physics, Amsterdam University Medical Centers University of Amsterdam the Netherlands

**Keywords:** cardiovascular, compressed sensing, flow quantitation, sampling strategies

## Abstract

**Introduction:**

Time‐resolved three‐dimensional phase contrast MRI (4D flow) of aortic blood flow requires acceleration to reduce scan time. Two established techniques for highly accelerated 4D flow MRI are k‐t principal component analysis (k‐t PCA) and compressed sensing (CS), which employ either regular or random k‐space undersampling. The goal of this study was to gain insights into the quantitative differences between k‐t PCA‐ and CS‐derived aortic blood flow, especially for high temporal resolution CS 4D flow MRI.

**Methods:**

The scan protocol consisted of both k‐t PCA and CS accelerated 4D flow MRI, as well as a 2D flow reference scan through the ascending aorta acquired in 15 subjects. 4D flow scans were accelerated with factor *R* = 8. For CS accelerated scans, we used a pseudo‐spiral Cartesian sampling scheme, which could additionally be reconstructed at higher temporal resolution, resulting in *R* = 13. 4D flow data were compared with the 2D flow scan in terms of flow, peak flow and stroke volume. A 3D peak systolic voxel‐wise velocity and wall shear stress (WSS) comparison between k‐t PCA and CS 4D flow was also performed.

**Results:**

The mean difference in flow/peak flow/stroke volume between the 2D flow scan and the 4D flow CS with *R* = 8 and R = 13 was 4.2%/9.1%/3.0% and 5.3%/7.1%/1.9%, respectively, whereas for k‐t PCA with R = 8 the difference was 9.7%/25.8%/10.4%. In the voxel‐by‐voxel 4D flow comparison we found 13.6% and 3.5% lower velocity and WSS values of k‐t PCA compared with CS with R = 8, and 15.9% and 5.5% lower velocity and WSS values of k‐t PCA compared with CS with R = 13.

**Conclusion:**

Pseudo‐spiral accelerated 4D flow acquisitions in combination with CS reconstruction provides a flexible choice of temporal resolution. We showed that our proposed strategy achieves better agreement in flow values with 2D reference scans compared with using k‐t PCA accelerated acquisitions.

## Graphical abstract

The goal of this study was to gain insights in the quantitative differences between compressed sensing and k‐t PCA‐derived aortic blood flow, especially for high temporal resolution compressed sensing 4D flow MRI. Random pseudo‐spiral sampling with compressed sensing reconstruction provides a more flexible choice of temporal resolution compared with regular sampling like k‐t PCA. We showed that our proposed strategy achieves better agreement in peak flow values with 2D reference scans compared with using k‐t PCA accelerated acquisitions.

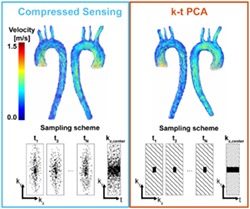

**Abbreviations**
AAo: ascending aorta
BA: Bland–Altman
BART: Berkeley Advanced Reconstruction Toolbox
CASPR‐Tiger: CArtesian trajectory with Spiral PRofile ordering and Tiny golden angle step for eddy current reduction
CPU: central processing unit
CS: compressed sensing
CS‐PI: compressed sensing and parallel imaging
DAo: descending aorta
ECG: electrocardiogram
FOV: field of view
G‐CASPR: golden‐step Cartesian acquisition with spiral profile order
GOCART: GOlden‐angle CArtesian randomized time‐resolved 3D MRI
GRAPPA: GeneRalized Autocalibrating Partially Parallel Acquisitions
k‐t PCA: k‐t principal component analysis
LoA: limits of agreement
MUSIC: multiphase, steady‐state imaging with contrast enhancement
MRI: magnetic resonance imaging
PC: phase contrast
PCA: principal component analysis
PC‐MRI: phase contrast magnetic resonance imaging
PI: parallel imaging
PROUD: PROspective Undersampling in multiple Dimensions
R: acceleration factor
RAM: random access memory
ROCK‐MUSIC: rotating cartesian K‐space ‐ MUSIC
ROI: region of interest
SEM: standard error of the mean
SENSE: sensitivity encoding
TE: echo time
TR: repetition time
4D flow MRI: time‐resolved three‐dimensional PC MRI
VDRad: variable density sampling and radial view ordering
VENC: velocity encoding
WSS: wall shear stress
XD‐GRASP: golden‐angle radial MRI with reconstruction of extra motion‐state dimensions using compressed sensing

## 1 INTRODUCTION

Cardiovascular phase contrast magnetic resonance imaging (PC‐MRI) is a well‐validated method to quantify pulsatile blood flow in the human aorta.^1^ Time‐resolved three‐dimensional PC MRI scans (4D flow MRI) are used to observe velocity or blood flow patterns and identify possible abnormalities such as flow jets or regurgitation.^2^ Moreover, advanced hemodynamic biomarkers can be derived, such as pulse wave velocity and wall‐shear stress (WSS). These biomarkers have shown to be valuable tools for risk assessment in aortic aneurysms, aortic plaques and stroke.^3^, ^4^

Unfortunately, 4D flow MRI has the inherent problem of requiring a long scan time. Depending on many factors, such as the field of view (FOV), spatial and temporal resolution, and also the breathing pattern and heart rate of the patient, the acquisition time for a 4D flow MRI scan can amount to 25 minutes or more.^1^, ^5^ Therefore, acquisition acceleration to reduce scan times to a clinically acceptable duration is imperative.

A commonly known technique to accelerate 4D flow MRI acquisitions is k‐t PCA,^6^ which exploits the time dependency in the 4D flow MRI data for acceleration. K‐t PCA allows for an 8‐fold acceleration and has shown great potential for clinical application in congenital heart disease.^7^, ^8^ In this technique, prospective ECG gating is combined with a highly ordered undersampling scheme in k‐t space at a predefined number of cardiac frames, which are reconstructed using principle component analysis (PCA).

Another promising method to speed up 4D flow MRI acquisitions is incoherent undersampling and compressed sensing (CS) reconstruction.^9^, ^10^ A flexible approach has been the use of pseudo‐radial or pseudo‐spiral acquisitions on a Cartesian grid, such as VDRad,^11^ GOCART,^12^ MUSIC^13^ and ROCK‐MUSIC,^14^ or G‐CASPR^15^ and CASPR‐Tiger.^16^ Pseudo‐spiral acquisitions in the k_y_/k_z_‐plane with a golden‐angle increment^17^ combined with retrospective binning of cardiac frames results in high spatiotemporal incoherency of subsequent cardiac frames, which is optimal for CS reconstruction. Furthermore, this sampling scheme together with retrospective binning provides the option of reconstructing the data in a variable number of cardiac frames.

The above strategies require modification of the scanner software to allow for a predefined undersampling trajectory compatible with cardiac gated sequences. At our institution (Amsterdam University Medical Centers, University of Amsterdam, the Netherlands) we have developed such a software patch, which we refer to as PROUD (PROspective Undersampling in multiple Dimensions). PROUD sampling is most similar to CASPR‐Tiger^16^ and incorporated variable density sampling^11^ as well as phase‐contrast imaging, but not self‐gating.

In the development and integration of 4D flow scans in the clinic, the issue arises as to which acceleration technique will be the most suitable. To address this, comparisons between different methods are needed to evaluate their performances, as well as their advantages and disadvantages.

In this study, we quantified and visualized aortic flow, velocity and WSS data derived from pseudo‐spiral CS accelerated 4D flow MRI using PROUD, as well as from k‐t PCA accelerated 4D flow MRI. Our purpose was to compare both methods with each other and with 2D flow MRI. Because PROUD CS has the flexibility of reconstructing at different temporal resolutions, a secondary aim was to investigate flow rate errors with increasing temporal resolution.

## 2 METHODS

### 2.1 Study design and subject cohort

Fifteen subjects (eight males and seven females) with an average age of 26.1 ± 3.5 years were included in the study. Each subject provided written informed consent prior to the start of the study. The study was waived by the local Medical Ethics Review Committee because the study deals with scan technique comparisons and does not involve patients. Scans were performed on a 3 T Philips Ingenia scanner using a 24‐channel torso coil (Philips Healthcare, Best, the Netherlands). After scout scans to locate the aorta, two 4D flow MRI scans covering the thoracic aorta were performed, either using k‐t PCA or our proposed pseudo‐spiral PROUD CS acceleration. The order of the 4D flow MRI scans was changed randomly per subject to avoid a systematic error due to the scan order. Furthermore, a single‐slice 2D flow MRI scan through the ascending aorta (AAo) and descending aorta (DAo) was acquired as a reference. Detailed scan parameters of all scans are given in Table 1 and were chosen according to those described in the consensus statement.^16^

**Table 1:** Detailed scan parameters of all scans

Parameter	4D flow k‐t PCA	4D flow PROUD CS	2D flow
FOV (FH x AP x RL) [mm]	315 x 275 x 60	315 x 275 x 60	8 x 350 x 301.4
ACQ voxel size (FH x AP x RL) [mm]	2.5 x 2.5 x 2.5	2.5 x 2.5 x 2.5	8 x 2.5 x 2.5
Recon voxel size (FH x AP x RL) [mm]	2.5 x 2.5 x 2.5	2.5 x 2.5 x 2.5	8 x 1.22 x 1.22
Slices (slice encoding direction)	24 (RL)	24 (RL)	1 (FH)
Cardiac frames	24	24	40
Acceleration factor (technique)	8 (k‐t PCA)	8 (CS)	2 (SENSE)
True acceleration factor (full k‐space/acquired k‐space)	6.92 ± 0.00	10.52 ± 0.22	2
TE/TR [ms]	2.1/4.2	2.1/3.9	2.5/4.1
Flip angle [degrees]	8	8	10
VENC [cm/s]	150	150	150
PC flow directions	FH, AP, RL	FH, AP, RL	FH
ECG‐gating	Prospective	Retrospective	Prospective
Respiratory compensation	Navigator	Navigator	Breath hold
Respiratory navigator gating window [mm]	7	7	‐
Nominal scan time [minutes: seconds]	05:45	04:25	00:12.7
Estimated scan time (with ~ 60% gating efficiency) [minutes: seconds]	09:35	07:22	00:12.7
Offline image reconstruction time [minutes]	10–15	14–18	‐

### 2.2 Pseudo‐spiral PROUD acquisition and CS reconstruction

**Figure 1:**
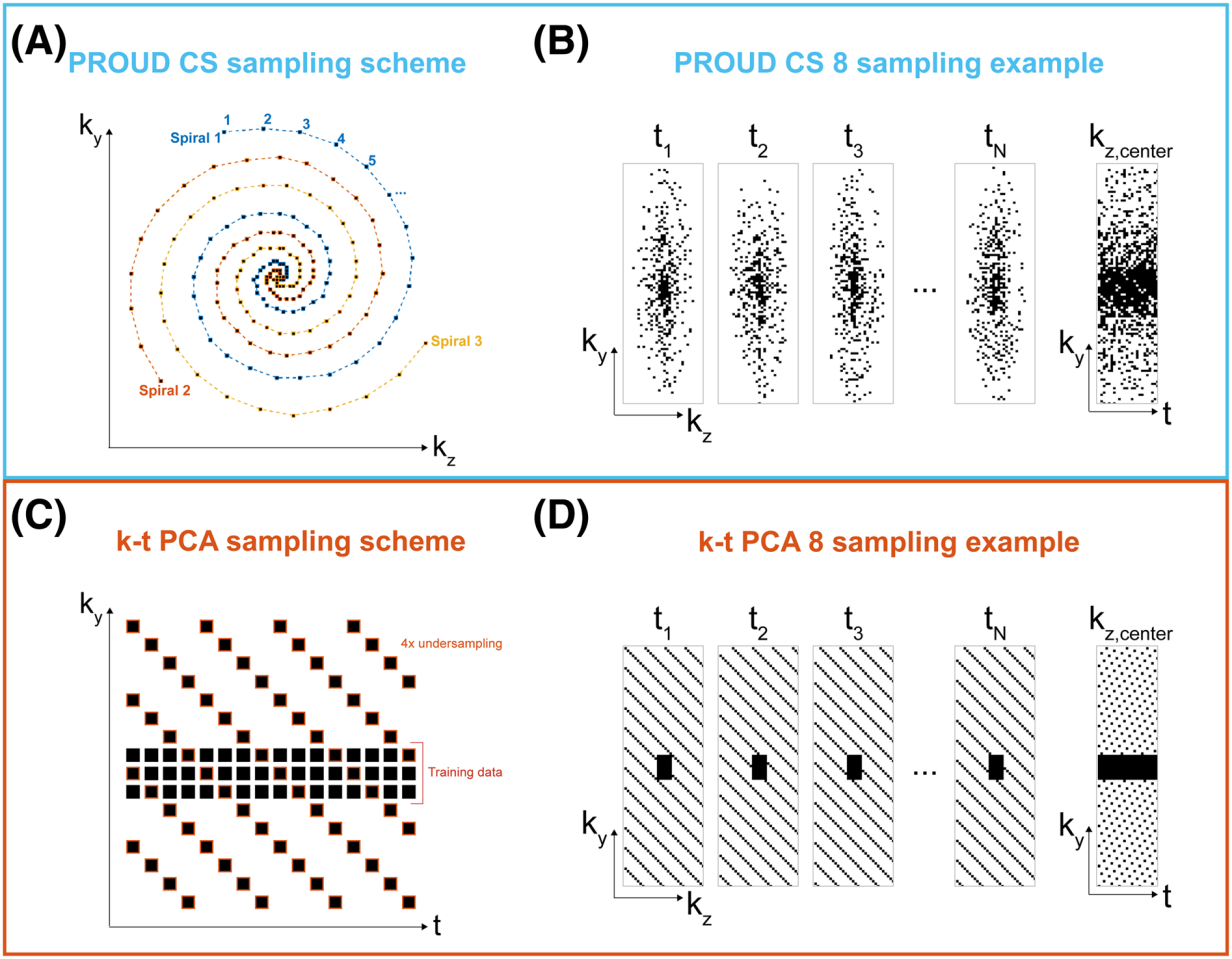
(A), The pseudo‐spiral PROUD CS sampling pattern consisted of multiple spirals. Consecutive spirals were turned by a golden angle (~ 137.51°). (B), Resulting sampling distributions for PROUD CS in spatial and time dimensions. (C), k‐t sampling pattern used for k‐t PCA was composed of a training set and an undersampled set, which were acquired simultaneously. (D), Sampling distributions for k‐t PCA in spatial and time dimensions

In this study we used a pseudo‐spiral k_y_/k_z_‐plane acquisition scheme designed for incoherent undersampling. The sampling trajectory list was calculated prior to the scans in MATLAB R2016a (MathWorks, Natick, MA, USA) and was passed to the scanner in the form of a text file. The shape of each outer‐in spiral arm can be described in the polar coordinates (*r*, ϕ) as

$$r\left(n\right)=\phi_{n}^{2};\phi_{n}=n\cdot \frac{2\pi \cdot T}{N}+\emptyset \;\left(n\in \left[0,N\right]\right),$$

where n is the index of the sampling location within each spiral arm, *N* is the total number of sampling locations (frequency encoding lines), and T is the number of spiral turns. In this study, N was 75 and T was 3. ∅ is the offset angle. For subsequent spiral arms, ∅ increases by a golden angle of ~ 137.51 degrees or 2.40 radians. Spirals were stretched to match the size of k‐space and gridded on Cartesian coordinates by rounding off to the nearest integer. The shapes of three consecutive spirals are illustrated in Figure 1A. The distance from the sampling locations to the k‐space center is changed quadratically along the spiral. Thus, the resulting total sampling has a variable density distribution with a denser sampling of the low‐frequency k‐space center and sparse sampling of high‐frequency peripheral k‐space (Figure 1B).

PROUD CS sampling overruled the scanner sampling on the basis of the desired sampling trajectory and was performed continuously without ECG triggering. The ECG signals were, however, used to store the acquisition time points within each cardiac cycle to allow for retrospective binning of different cardiac frames. Importantly, due to the physiological heart‐rate variability, a random filling of k‐t space was created with a mean undersampling factor of ~ 8 per cardiac frame (Figure 1B). Oversampling of the same k‐t‐space coordinate was avoided in real time by checking whether a k‐t space coordinate had already been scanned and, if this was the case, skipping this acquisition and continuing with the next k‐space trajectory coordinate.

The acceleration factor for each PROUD scan, *R*_*proud*_, was calculated retrospectively based on a theoretical fully sampled scan:

$$R_{\text{proud}}=\frac{N_{\text{full}}}{N_{\text{undersampled}}}=\frac{N_{\mathrm{ky}}*N_{\mathrm{kz}}*N_{\mathrm{card}}*\pi /4}{N_{\text{proud}}},$$

where *N*_*ky*_, *N*_*kz*_ and *N*_*card*_ are the number of phase‐encoding steps, number of slices and number of cardiac frames, respectively, for a fully sampled scan, and *N*_*proud*_ is the total number of PROUD sampling points of all pseudo‐spirals. The factor *π*/4 describes the absence of sampling at the outer edges of k‐space (elliptical k‐space shutter^18^).

The PROUD scans were primarily reconstructed in 24 cardiac frames, resulting in an acceleration factor of 8 (hereafter called PROUD CS 8), and also in 40 cardiac frames, resulting in an acceleration factor of 13 (hereafter called PROUD CS 13). Additional reconstructions were made with 12, 27, 35 and 60 cardiac frames.

However, the true acceleration factor, *R*_*true*_, which is defined as the number of samples in the full k‐space divided by the number of points in the undersampled acquired k‐space, is *π*/4 times higher and can vary per cardiac frame as well as per subject. The mean *R*_*true*_ over all cardiac frames and all subjects is provided in Table 1. CS reconstructions were performed on a Linux server in MATLAB using MRecon (Gyrotools, Zurich, Switzerland) in combination with the Berkeley Advanced Reconstruction Toolbox (BART)^19^; the server allowed the use of eight Intel Xeon Gold 6132 CPUs at 2.6 GHz and up to 300 GB RAM. A nonlinear parallel imaging and compressed sensing (PI‐CS) reconstruction was performed according to

$$\underset{\;m}{\arg\;\min}\left\{{\left\|F_{U}\mathrm{Sm}-y\right\|}_{2}+\lambda \;{\left\|\mathrm{Tm}\right\|}_{1}\right\}$$

using a sparsifying total variation operator in time T. F_U_ denotes the undersampling Fourier operator, S the coil sensitivity maps, y the measured k‐space data, and m the reconstructed image data. While the left term ensures data consistency, the right term enforces sparsity, regularized by parameter λ. The reconstruction was performed with a regularization parameter of λ = 0.001 and *i* = 20 iterations. The applied reconstruction was the result of an L‐curve^20^ analysis for one slice of one subject in combination with 2D flow comparison. The analysis included the regularization constraints wavelet transformation in space and/or total variation in space and/or time, various regularization parameters λ ranging from 0.0001 to 0.5, and various numbers of iterations i ranging from 20 to 50.

### 2.3 **K**‐t PCA acquisition and reconstruction

For k‐t PCA, the k‐t space was uniformly undersampled on a Cartesian grid^6^ using FlowPatch (Gyrotools) with a k‐t acceleration factor of *R* = 8 (hereafter called k‐t PCA 8). Prospective ECG‐gating was used with an acquisition of 24 cardiac frames. Training data, consisting of 11 center phase‐encoding lines for each time point, were collected simultaneously (Figure 1C,D) resulting in a lower true acceleration factor *R*_*true*_ (Table 1).

K‐t PCA 4D flow data were reconstructed with CRecon (Gyrotools) on a Windows computer with eight Intel Xeon E5–1620 CPUs at 3.5 GHz and up to 16 GB RAM. To avoid temporal blurring, the k‐t regularization factor was optimized by 2D peak flow comparison of one exemplary dataset. A value of λ = 0.2 resulted in the best image quality and the lowest flow bias, and was thus used for all subsequent k‐t PCA reconstructions.

### 2.4 Flow analysis

Following the reconstruction, a phase‐contrast MRI angiogram (PC‐MRA) was created by voxel‐wise multiplication of the phase‐contrast magnitude images with the absolute velocity, and subsequently averaged over all cardiac frames.^21^ PC‐MRA data were used for segmentation of the aorta anatomy in Mimics Research 21.0 (Materialise, Leuven, Belgium).^22^ Segmentations were conducted for all 4D flow MRI sequences separately.

For 2D analysis, 4D flow MRI scans were resliced to the slice position of the 2D flow MRI scan in GTFlow (Gyrotools). ROIs of the AAo and DAo were drawn in the 2D flow MRI scan as well as in the resliced 4D flow MRI images. All ROIs were drawn in the images at the time of peak flow. To match the highest temporal resolution of 40 cardiac frames, all velocity and flow measurements of lower temporal resolution were interpolated by a shape‐preserving piecewise cubic interpolation.

For 3D analysis, WSS at the peak velocity time frame was calculated in MATLAB, as previously described.^23^ For voxel‐wise comparisons of velocity and WSS, datasets were registered by a rigid transformation followed by nearest neighbor interpolation. Additionally, directional differences in velocity and WSS vectors were assessed by comparing angular difference distributions.^22^, ^24^

### 2.5 Statistical analysis

For 2D analysis, the flow of the 4D flow scans were compared with the 2D flow scan as a reference using Bland–Altman (BA) analysis and orthogonal regression plots.^25^ Differences in the peak flow and stroke volume—the integral of the measured flow over the cardiac cycle—between each of the 4D flow scans with the 2D scan were reported in ml/s or ml, respectively, or in a percentage difference. Confidence limits are reported as mean and standard error of the mean (SEM). Significance was tested for peak flow and stroke volume by a two‐sided t‐test with 0.05 used as the cutoff for significance.

For 3D analysis, velocity and WSS values were compared by voxel‐by‐voxel BA analysis and orthogonal regression.

For all analyses, mean differences and limits of agreement (LOA) were reported for the BA analysis and slope, intercept and Pearson's r for the orthogonal regression analysis. Relative difference measures were reported in percentages to the mean of all values.

## 3 RESULTS

### 3.1 2D analysis

**Figure 2:**
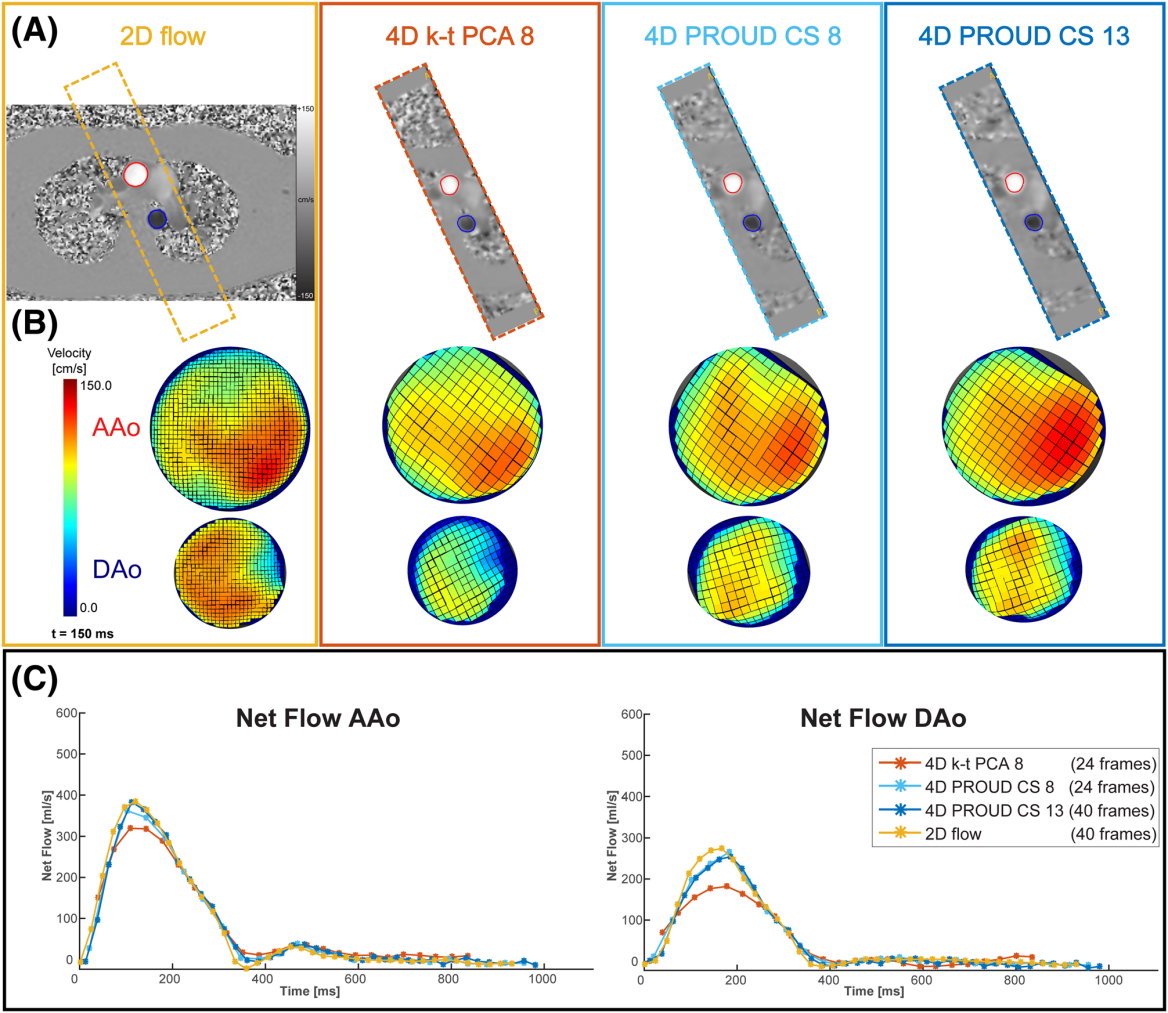
(A), Exemplary through‐plane velocity images of the 2D flow MRI as well as accelerated 4D flow MRI scans. ROIs are depicted in the ascending aorta (AAo, red) and descending aorta (DAo, blue). (B), The corresponding velocity scalar maps. (C), The corresponding flow curves for the four flow scans (AAo left and DAo right)

An example of a 2D flow image with the corresponding images of the three accelerated 4D flow scans is shown in Figure 2A. Color‐coded velocity profiles in the ROIs in the AAo and DAo are shown in Figure 2B with good agreement of the velocity profiles between the accelerated 4D flow and 2D flow scans as a reference. Flow values of all four scans during the cardiac cycle are shown in Figure 2C for the AAo and DAo. Here, it can be seen that for both the AAo and the DAo, PROUD CS flow curves were better matched with the 2D flow curve compared with k‐t PCA.

**Figure 3:**
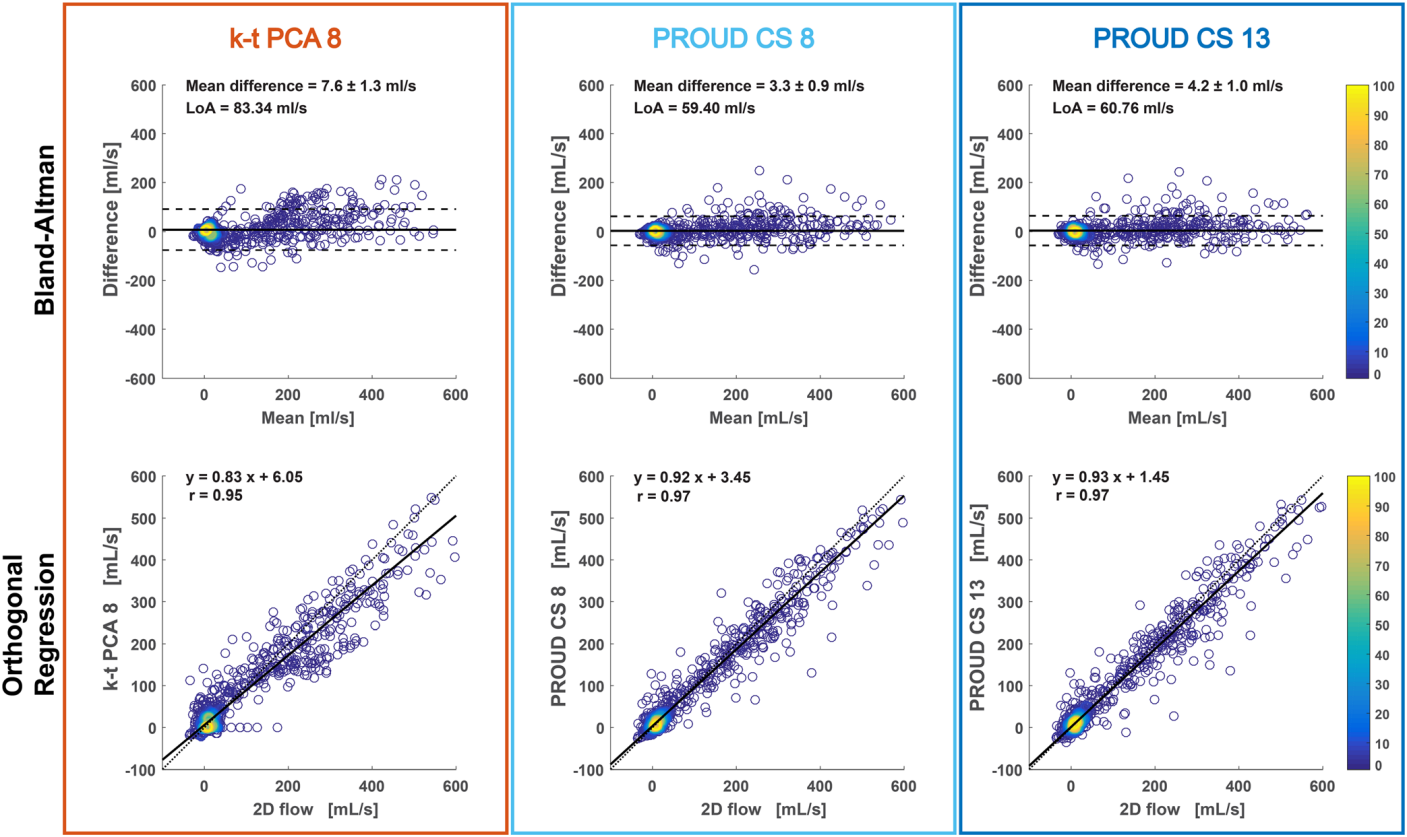
Bland–Altman plot (top) and orthogonal regression (bottom) of flow for the accelerated 4D flow MRI scans compared with the 2D flow MRI scan combined for all time frames and subjects. The color bar indicates the number of touching circles

Results for BA and orthogonal regression analyses are given in Figure 3 and Table 2. Looking at the mean difference as well as difference relative to the mean, it can be seen that all 4D flow scans underestimated flow compared with a 2D flow scan. K‐t PCA 8 scans showed a larger underestimation than PROUD CS 8 and PROUD CS 13. Additionally, LoA in the BA plots were smaller for PROUD CS 8 and PROUD CS 13 than for k‐t PCA 8. Looking at the orthogonal regression, it can be seen that 4D flow scans underestimated peak systolic flow compared with a 2D flow scan. The slope of the regression equation was smaller for k‐t PCA 8 compared with PROUD CS 8 and PROUD CS 13, showing that k‐t PCA 8 scans had a larger underestimation. Nevertheless, the Pearson correlation coefficients for k‐t PCA 8, PROUD CS 8 and PROUD CS 13 indicated a strong correlation.

**Table 2:** 2D flow scan compared with corresponding slice of accelerated 4D flow scans

Accelerated 4D flow MRI vs. 2D flow MRI						
Bland–Altman				Orthogonal regression		Correlation
4D flow scan	Mean difference (±SEM) [ml/s] (%)	Median of the difference relative to the mean [%]	LoA [ml/s]	Slope	Intercept [ml/s]	r
k‐t PCA 8	−7.6 ± 1.3 (9.7)	−10.3	83.3	0.83	6.06	0.95
PROUD CS 8	−3.3 ± 0.9 (4.2)	−5.0	59.4	0.92	3.45	0.97
PROUD CS 13	−4.2 ± 1.0 (5.3)	−4.1	60.8	0.93	1.45	0.97

**Figure 4:**
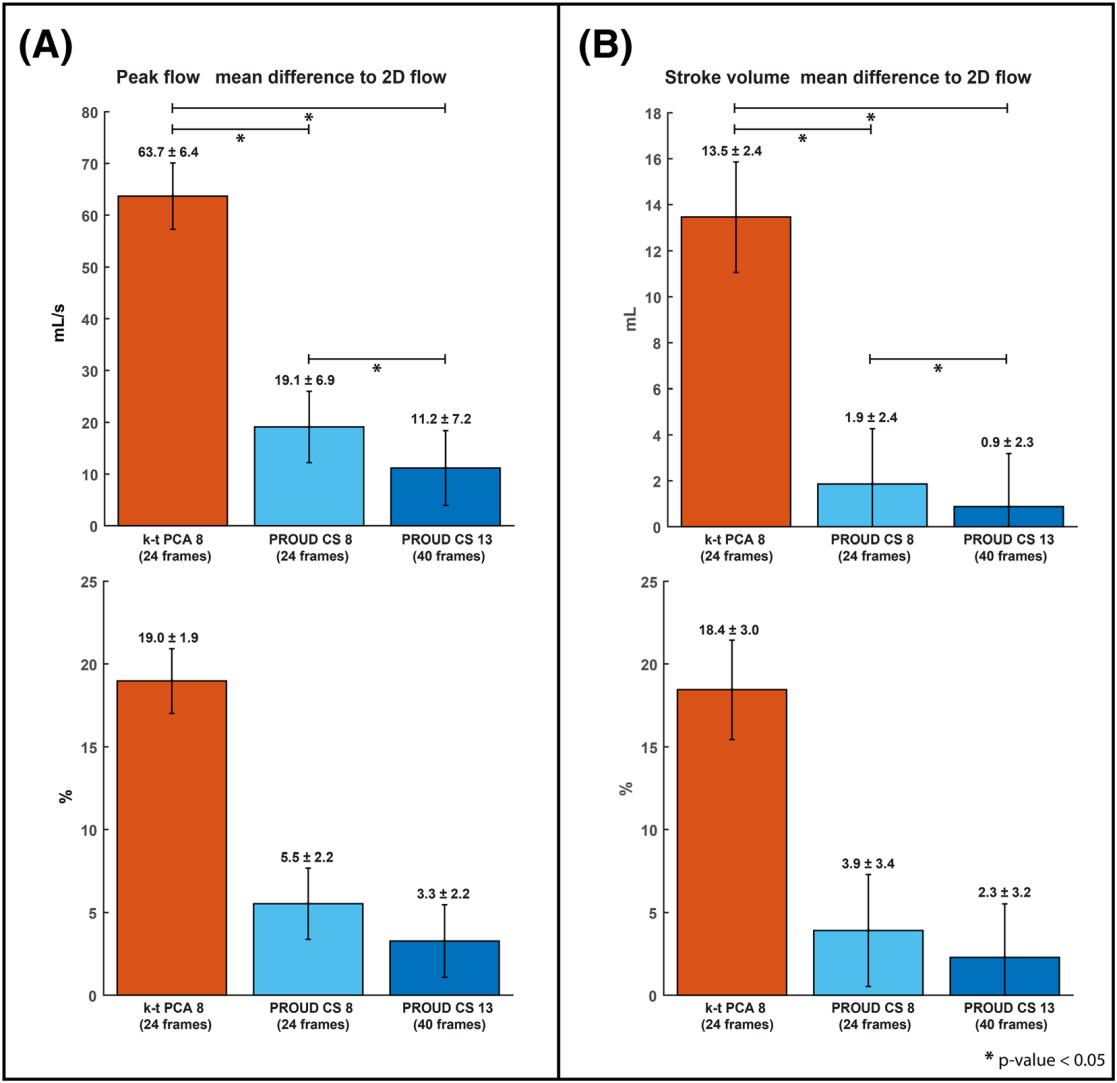
Bar charts of the mean difference (±SEM) between accelerated 4D flow MRI and 2D flow MRI for the peak flow (A), and the stroke volume (B),. The same charts in percentages are shown at the bottom

Furthermore, the mean difference of the stroke volume and the peak flow per 4D flow MRI scan were compared with the 2D flow scan (Figure 4). Higher deviations to 2D flow were found for k‐t PCA than for PROUD CS. Stroke volume differences between the 4D flow and 2D flow scans were 13.5 ± 2.4 mL for k‐t PCA 8, 1.9 ± 2.4 mL for PROUD CS 8 and 0.9 ± 2.3 mL for PROUD CS 13. Peak flow differences to the 2D flow were 63.7 ± 6.4 mL for k‐t PCA 8, 19.1 ± 6.9 mL for PROUD CS 8 and 11.2 ± 7.2 mL for PROUD CS 13. All stroke volume differences as well as all peak flow differences were significantly different to each other. Mean difference of the peak flow and the stroke volume between 2D flow MRI and PROUD CS reconstructed with 12, 24, 27, 30, 35, 40 and 60 cardiac frames are shown in Figure S1. Stroke volume and peak flow differences to 2D flow decrease with an increasing number of cardiac frames. PROUD CS 13 with 40 cardiac frames proves to be the best option as the stroke volume difference increases again from 40 to 60 cardiac frames. An example magnitude and PC image sequence for the three different 4D flow datasets, k‐t PCA 8, PROUD CS 8 and PROUD CS 13, are shown in Figure S2.

### 3.2 3D analysis

**Figure 5:**
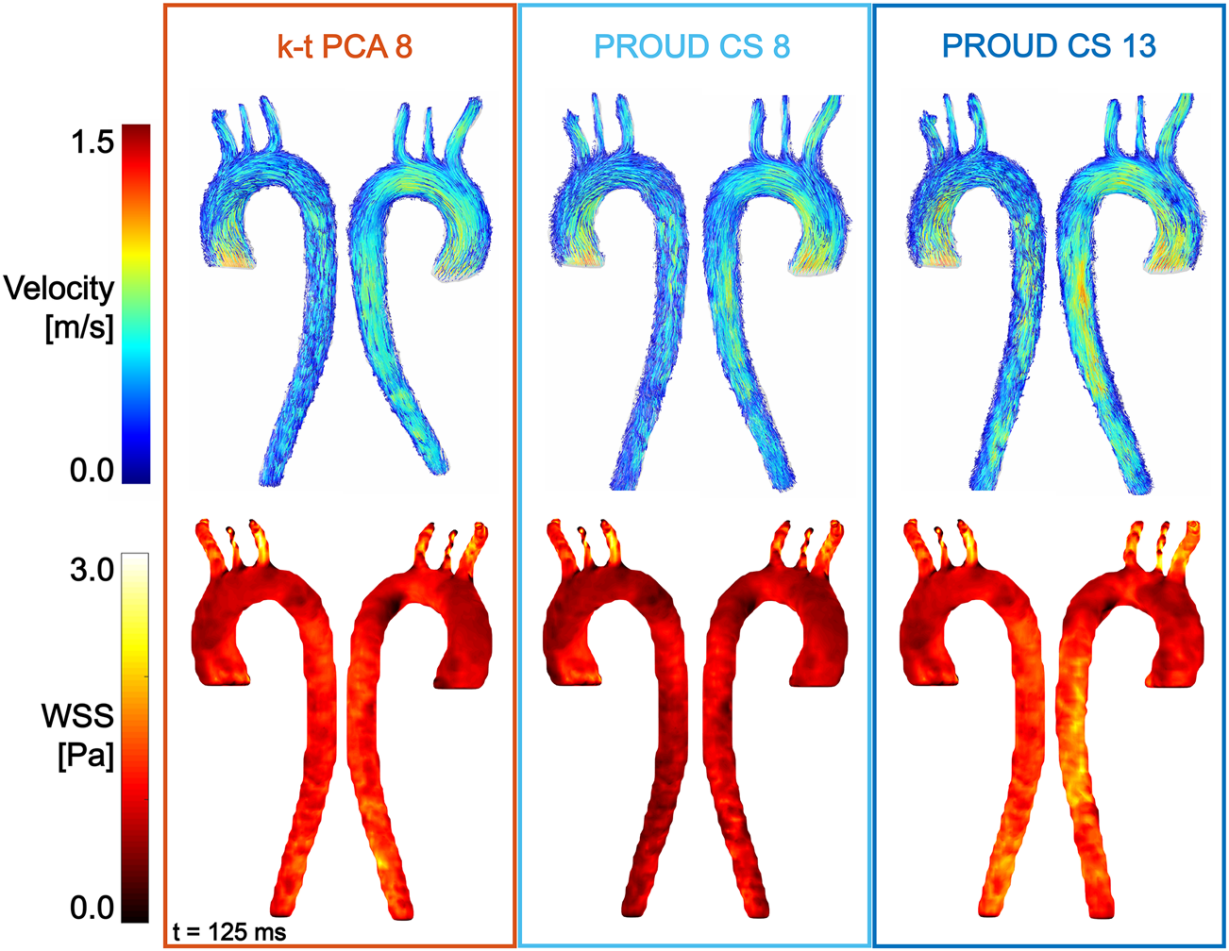
Example visualization of peak systolic flow path lines (top) and wall‐shear stress (WSS) (bottom) for the different 4D flow scans of one subject. [Correction added on 27 January 2020, after first online publication: figure 5 has been updated]

Representative 4D flow visualizations in the form of path lines as well as WSS maps are shown in Figure 5. The path line comparison shows similar flow patterns for all 4D flow scans as well as similar velocity and WSS magnitude values.

**Figure 6:**
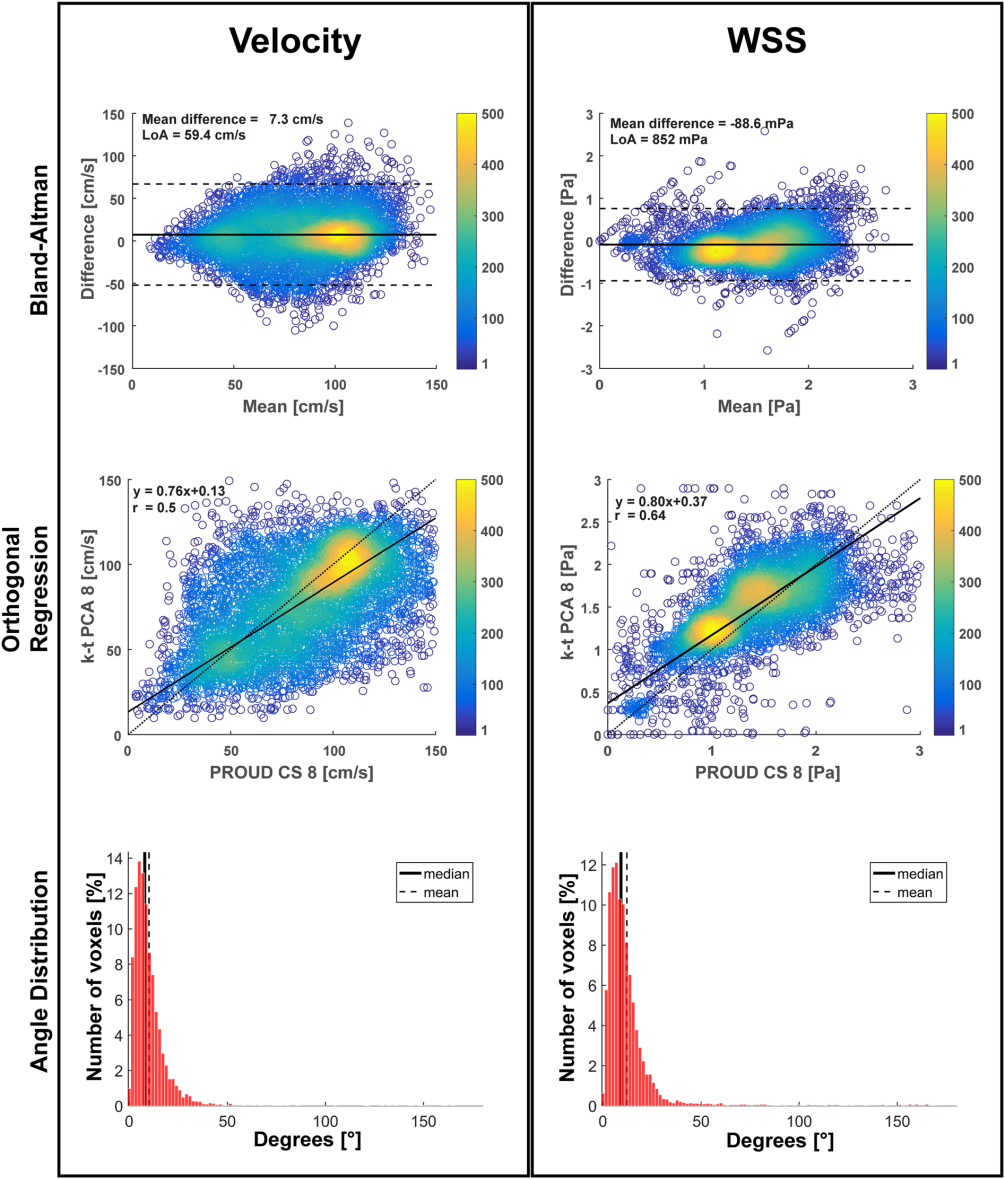
Comparison of peak systolic velocity and wall‐shear stress (WSS) for one subject between k‐t PCA 8 and PROUD CS 8 scans showing Bland–Altman (top), orthogonal regression (middle) and angle distribution analyses (bottom). The color bar indicates the number of touching circles

Results from voxel‐by‐voxel BA, orthogonal regression and angle distribution analysis for velocity and WSS are shown in Figure 6 for an example dataset. Orthogonal regression showed an underestimation of velocity and WSS for k‐t PCA 8 compared with PROUD CS 8, especially for higher values. The results of velocity and WSS comparisons of all subjects combined are listed in Table 3.

**Table 3:** 3D analysis: Results of Bland–Altman, orthogonal regression and angle distribution analyses for velocity and wall shear stress (WSS) vectors between the different 4D flow scans

4D flow comparison						
	Bland–Altman		Orthogonal regression		Correlation	Angle distribution
Velocity:	Mean difference (±SEM) [cm/s] (%)	LoA [cm/s]	Slope	Intercept [m/s]	r	Median [°]
PROUD CS 8 vs. k‐t PCA 8	9.40 ± 0.08 (13.6)	51.8	0.84	0.03	0.51	9.2
PROUD CS 13 vs. PROUD CS 8	1.43 ± 0.04 (1.9)	23.8	0.93	0.04	0.92	4.8
PROUD CS 13 vs. k‐t PCA 8	11.10 ± 0.08 (15.9)	54.4	0.73	0.09	0.51	9.6
WSS:	[mPa] (%)	[mPa]		[pa]		
PROUD CS 8 vs. k‐t PCA 8	41.0 ± 1.5 (3.5)	850	0.92	0.05	0.66	10.1
PROUD CS 13 vs. PROUD CS 8	17.8 ± 0.8 (1.5)	444	0.94	0.06	0.92	4.7
PROUD CS 13 vs. k‐t PCA 8	65.0 ± 1.6 (5.5)	904	0.84	0.14	0.64	10.5

Table 3 shows that mean differences in velocity and WSS values, as well as LoA, Pearson's r and median angle, were similar for k‐t PCA versus PROUD 8, and k‐t PCA versus PROUD 13. Furthermore, agreement was strongest for PROUD 8 versus PROUD 13. This result indicated that, when using PROUD CS 4D flow, peak systolic cardiac frames can be resolved correctly when reconstructing data into 40 instead of 24 cardiac frames.

## 4 DISCUSSION

In this study, we investigated the performance of pseudo‐spiral PROUD CS 4D flow MRI in healthy aorta (*R* = 8). CS reconstruction provided the option of retrospectively increasing the temporal resolution from 24 up to 40 cardiac frames (*R* = 13) without a penalty in flow, velocity and WSS quantification. Results were compared with 2D flow scans and k‐t PCA accelerated 4D flow MRI (R = 8). k‐t PCA and PROUD CS reconstructions resulted in comparable velocity and flow measurements.

Compared with time‐resolved 2D flow scan as a reference, a systematic underestimation of flow for all 4D flow scans was found, but PROUD CS scans showed lower differences and variability than k‐t PCA. The same observation of a systematic underestimation was found when comparing stroke volume and peak flow between 4D flow and 2D flow scans as reference. Here, peak flow and stroke volume differences to the 2D flow scan showed significantly lower biases for both PROUD CS scans compared with k‐t PCA 8, with the smallest difference observed for PROUD CS 13. This demonstrates that a high temporal resolution is favorable for better peak flow measurements (ie, 40 cardiac frames). This trend is especially highlighted in Figure S1, which showed that reconstructions of the same scan with high temporal resolution, or a high number of cardiac frames, respectively, led to small peak flow and stroke volume differences. Reconstructions with fewer cardiac frames resulted in larger biases. The larger stroke volume difference for PROUD CS with 60 cardiac frames might be a result of the comparison with a 2D reference scan that contains fewer cardiac frames. Furthermore, a higher temporal resolution requires a higher acceleration factor, which will come at the cost of decreased accuracy in the derived flow parameters. It depends on the type of application how this tradeoff between scan time and accuracy should be weighted.

Voxel‐wise comparison of peak systolic 3D velocity and WSS showed good correspondence between k‐t PCA and both PROUD CS 4D flow MRI reconstructions and indicated that temporal resolution can be retrospectively increased without a penalty in hemodynamic quantification. However, higher acceleration results in a larger magnitude image penalty, which is visibly more pronounced in the magnitude images for PROUD CS 13 (ie, Figure S2), but we considered this acceptable because the velocity images contain the important information. Small artifacts were seen in the velocity images as well, which may become problematic for slow flow or regurgitant flow. However, for fast forward flow, which most aortic 4D flow MRI studies focus on, this penalty is smaller and tolerable. Both PROUD CS and k‐t PCA 4D flow‐MRI are able to measure velocity and flow‐derived values such as WSS in the aorta. Even although our study showed that in 4D flow of the aorta PROUD CS offers some distinct advantages over k‐t PCA—specifically, missing k‐space lines could be compensated for in the reconstruction for CS but not for k‐t PCA, the temporal resolution could be changed for PROUD CS but not for k‐t PCA, and higher temporal resolution led to smaller flow measurement errors in PROUD CS—it is very difficult to generalize these findings to other vascular regions and pathological cases. PROUD CS acceleration benefits from slow temporal variations and spatial sparsity, whereas k‐t PCA exploits a limited number of temporal base functions for acceleration, which can also be considered a type of signal sparsity. For different vascular territories, flow patterns or pathological flow, one or the other type of sparsity might prove more effective to accelerate the acquisition.

The ability to retrospectively change the temporal resolution of the pseudo‐spiral PROUD 4D flow scans allows for more flexibility compared with k‐t PCA scans, in which the acceleration factor and the temporal resolution must be defined prior to the scan start. Additionally, for k‐t PCA, the number of cardiac frames must be a multiple of the acceleration factor.^6^ Also, the nonuniform sampling of the PROUD CS scans has no concern with incomplete scans, or aborts in general. That advantage can be seen in the case of a subject in which the PROUD CS scan ended at around only 50% scan percentage, but where data reconstruction was still possible (Figure S3). Moreover, in comparison with the AAo peak flow of the 2D flow scan, the incomplete PROUD CS scans showed lower differences than the complete k‐t PCA scan with *R* = 8. Therefore, PROUD CS has the important advantage of being able to reconstruct incomplete data that still result in usable 4D flow MRI data. Reconstruction times were in the range of 10 to 15 minutes for PROUD CS and in the range of 14 to 18 minutes for k‐t PCA.

Compared with other studies employing k‐t or CS acceleration for 4D flow MRI, we found similar results. Recently, Peper et al,^26^ who also used pseudo‐spiral Cartesian imaging with CS reconstruction, found that 4D flow MRI can be accelerated by up to *R* = 20 with an acceptable underestimation of less than 10%. Neuhaus et al,^27^ who used CS accelerated 4D flow MRI with R = 8, reported a peak flow difference of 4.6 ± 25.2 ml/s. Bollache et al^28^ found a peak flow difference of −4.2% to 3% (k‐t acceleration with *R* = 5), Knobloch et al^29^ found reduced peak velocities of 4.9% ± 7% (k‐t PCA with R = 8), and Giese et al^7^ found a peak flow underestimation of 5.1% ± 7.5% (k‐t PCA with R = 8). It was not possible to compare PROUD CS with k‐t PCA scans with a higher acceleration factor than 8. For instance, Knobloch et al^29^ have defined a k‐t PCA factor of 8 as the upper limit, and Pedersen et al^6^ defined R = 8 as a good tradeoff considering image quality and acceleration. Improvements for k‐t PCA have been published, such as k‐t sPCA^29^ and k‐t PCA+,^30^ where the most promising improvement was the spatial compartment dependent temporal basis function, which showed errors in stroke volume smaller than 5% for 16‐fold acceleration. CS reconstructions with subspace constraint based on PCA or equivalently low rank constraint also exist,^31^ but these were not used in this study. For CS on the other hand, studies showed that acceleration higher than a factor of 8 is feasible.^32^, ^33^ Ma et al^32^ showed peak flow differences to conventional 4D flow (GRAPPA R = 2) of −12.6% (AAo) and − 3.2% (DAo) for CS accelerated 4D flow MRI by a factor of *R* = 12.8. Compared with this study, we found a smaller peak flow difference for PROUD CS 13 compared with the reference of 7.1% for both ROIs combined. The smaller peak flow difference might be explained by the higher number of cardiac frames provided for PROUD CS 13 compared with the scan used in Ma et al.^32^ Interestingly, for intracranial applications, Liu et al^33^ showed that a higher acceleration factor for CS reconstruction in combination with higher temporal resolution can be used to increase accuracy. In their study, peak velocity differences compared with the reference (GRAPPA R = 2) could be reduced from −3.83 cm/s for *R* = 4 with 72 ms temporal resolution to −1.72 cm/s for R = 12 with 24 ms temporal resolution. This is similar to observations found in this study.

This study has some limitations. One possible explanation for the mentioned k‐t PCA flow underestimation is the challenge of the k‐t regularization strength selection. On the one hand, a too‐high value will smooth the flo*w*/*v*elocity over the cardiac cycle, but, on the other hand, a too‐low value will show more image artifacts, such as temporal blurring or flattening of the flow curves. As no optimal regularization strength was available, efforts have been made to choose the best regularization strength. Therefore, as for the CS reconstruction regularization parameter λ, we optimized the k‐t regularization strength for one‐subject reconstruction by comparison with the 2D flow scan, and used these settings for the entire study. Of course, optimization per subject would possibly lead to better results, but that approach is not feasible, especially for patient care, and was therefore not used.

For PROUD CS sampling, we neither explicitly measured the effects of eddy currents nor optimized our sampling scheme with respect to them. However, in this study we used a Cartesian T1‐weighted spoiled gradient‐echo acquisition, for which eddy currents do not lead to severe image artifacts.^34^

Another limitation is that in the 2D analysis, the interpolation of 24 to 40 cardiac phases could have caused additional differences in quantification. Another possibility for discrepancies in quantification is the difference in spatial resolution between 2D flow and 4D flow MRI. Also, the 2D flow MRI scan was acquired in a breath hold, whereas the 4D flow MRI scans were acquired in free breathing with respiratory gating. Therefore, the 2D flow scan can be considered as motion artifact‐free. By contrast, motion artifacts are reduced in the 4D flow scans due to the respiratory gating but cannot be eliminated. Moreover, increased intrathoracic pressure during breath hold is associated with flow and stroke volume decrease, which can be another reason for differences between 2D flow and 4D flow MRI.^35^, ^36^ Finally, the difference in peak systolic segmentation for the 4D flow scans in contrast to the time‐resolved segmentation of the 2D flow scans are another possible source of errors.^37^

Additional studies can make use of PROUD CS acceleration as a valuable tool in clinical research to speed up 4D flow acquisitions. The scope of PROUD CS includes any type of 4D flow measurements in various patient anatomies. A useful next step would be to compare the proposed PROUD CS method with a self‐gating approach such as XD‐GRASP,^38^ or to implement self‐gating for PROUD CS, as was demonstrated in related Cartesian 5D flow acquisitions.^14^, ^39^

## 5 CONCLUSION

In conclusion, both k‐t PCA and pseudo‐spiral PROUD CS demonstrated their ability to accelerate 4D flow measurements. We found that in this setting, PROUD CS was slightly superior compared with k‐t PCA as, among others, peak flow values were less underestimated in comparison with the 2D flow scan as a reference. Moreover, PROUD CS acquisition has the additional benefit of changing the temporal resolution during reconstruction. We showed that this advantage offers greater reconstruction versatility and led to even higher accuracy in hemodynamic measurements when reconstructing the data at a higher temporal resolution.

## FUNDING INFORMATION

Stichting voor de Technische Wetenschappen, grant 13928.

## Supporting information

Figure S1 Bar charts of the mean difference (±SEM) between accelerated 4D flow MRI and 2D flow MRI for the peak flow (A) and the stroke volume (B). The same charts in percentage are shown at the bottom.

Figure S2 Example magnitude and PC image sequence for the three different 4D flow data sets k‐t PCA 8, PROUD CS 8, and PROUD CS 13 showing a center slice in sagittal view over time.

Figure S3 Example magnitude and PC images for the three different 4D flow data sets k‐t PCA 8, PROUD CS 16, and PROUD CS 26 of the excluded subject for which the PROUD CS scan was incomplete around 50% scan time, resulting in PROUD CS acceleration factors of 16 and 26. The yellow line indicates the slice position of the 2D flow scan. Corresponding flow curves are depicted at the bottom.
