# Supplementary figures and images for: Pseudo‐spiral sampling and compressed sensing reconstruction provides flexibility of temporal resolution in accelerated aortic 4D flow MRI: A comparison with k‐t principal component analysis
Source: NMR Biomed. 2020 Jan 20;33(4):e4255. doi: 10.1002/nbm.4255 (PMC7079056; doi:10.1002/nbm.4255)

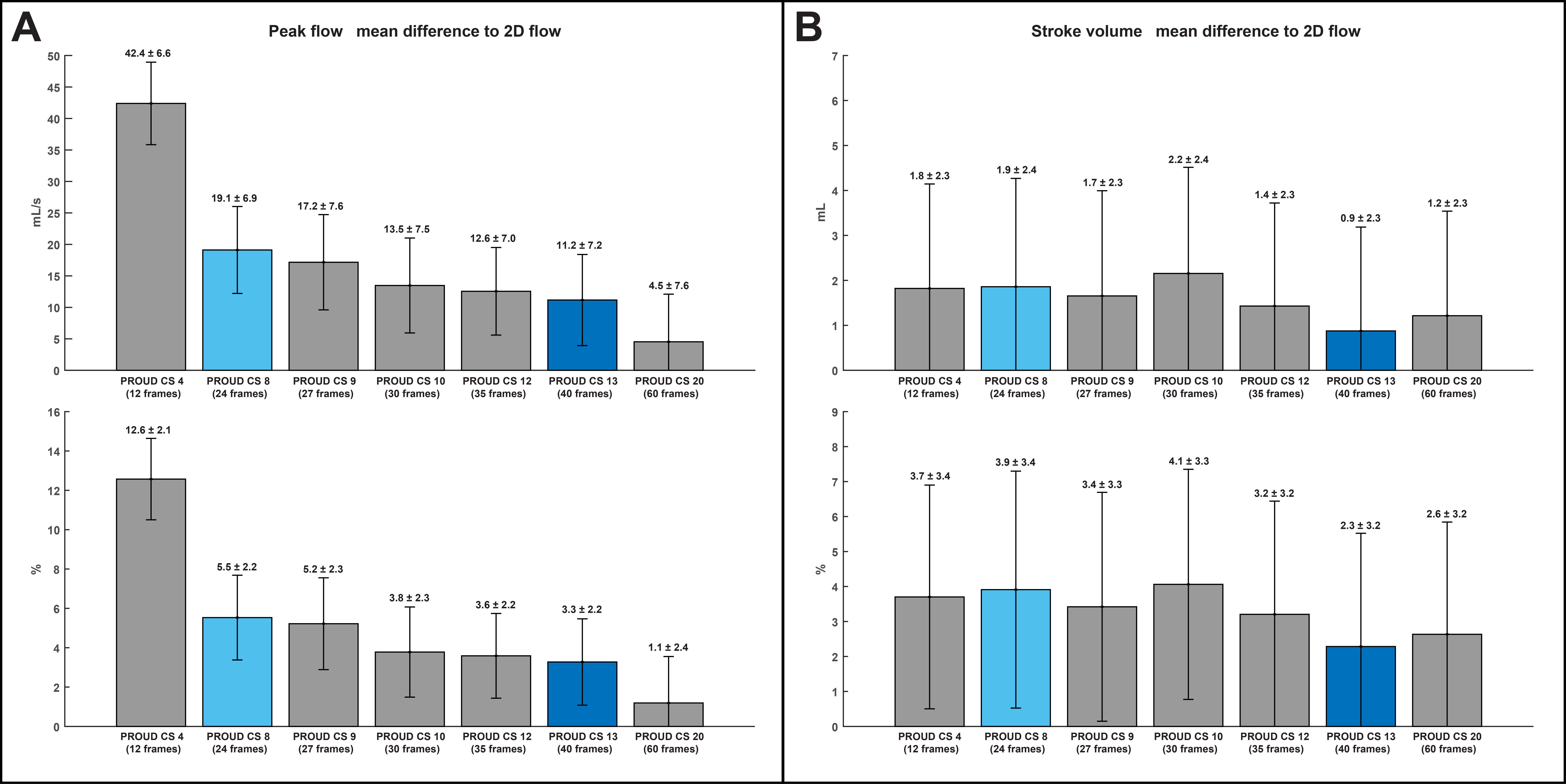

Supplement: Supplementary file 1 — Figure S1 Bar charts of the mean difference (±SEM) between accelerated 4D flow MRI and 2D flow MRI for the peak flow (A) and the stroke volume (B). The same charts in percentage are shown at the bottom. [file NBM-33-e4255-s001.tif]

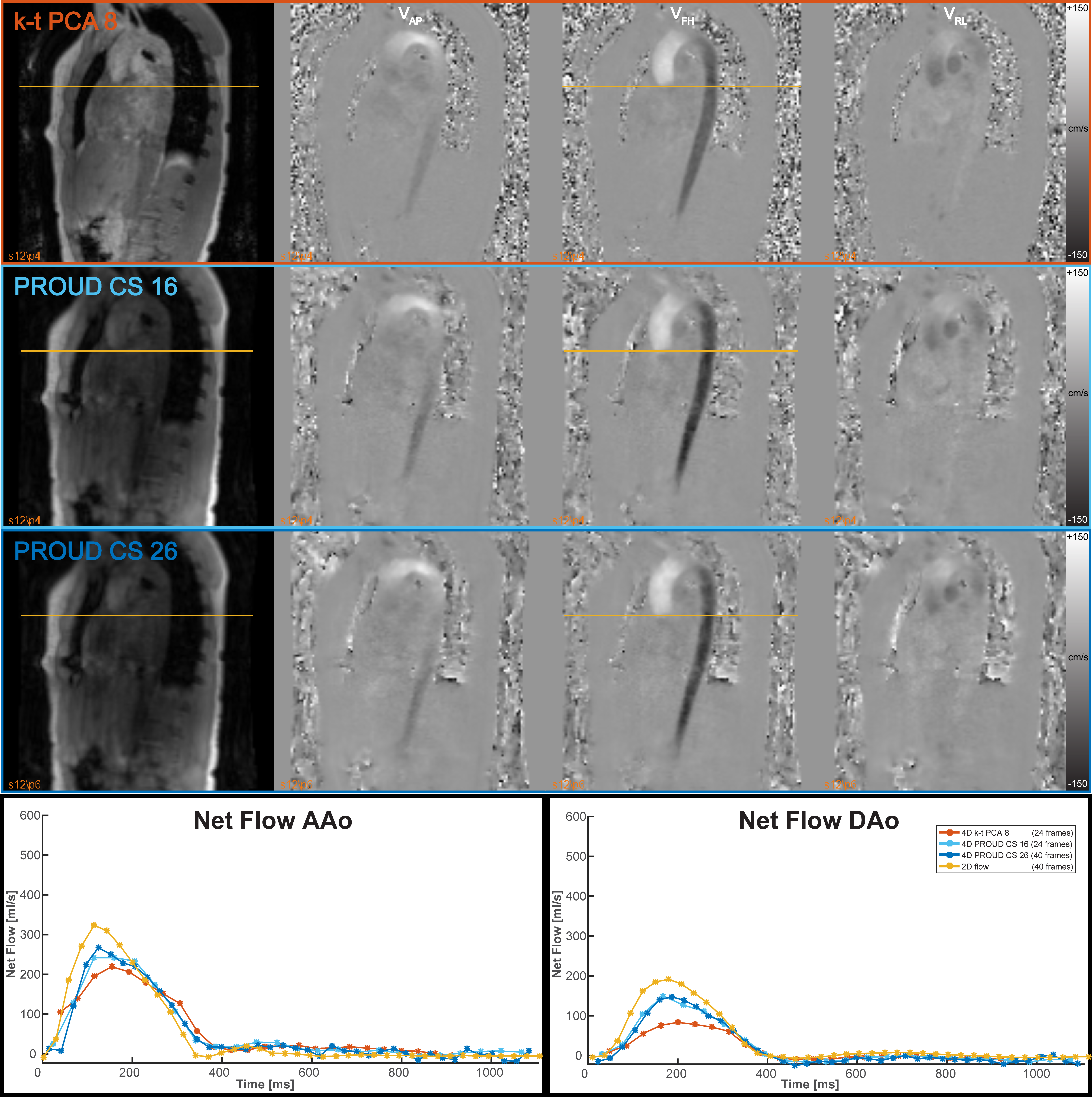

Supplement: Supplementary file 3 — Figure S3 Example magnitude and PC images for the three different 4D flow data sets k‐t PCA 8, PROUD CS 16, and PROUD CS 26 of the excluded subject for which the PROUD CS scan was incomplete around 50% scan time, resulting in PROUD CS acceleration factors of 16 and 26. The yellow line indicates the slice position of the 2D flow scan. Corresponding flow curves are depicted at the bottom. [file NBM-33-e4255-s003.tif]
